# A Cdk4/6-dependent phosphorylation gradient regulates the early to late G1 phase transition

**DOI:** 10.1038/s41598-021-94200-w

**Published:** 2021-07-19

**Authors:** Manuel Kaulich, Verena M. Link, John D. Lapek, Yeon J. Lee, Christopher K. Glass, David J. Gonzalez, Steven F. Dowdy

**Affiliations:** 1 Department of Cellular and Molecular Medicine, University of California San Diego, La Jolla, CA USA; 2 Institute of Biochemistry II, Goethe University, Frankfurt am Main, Germany; 3 Department of Pharmacology, University of California San Diego, La Jolla, USA; 4 Skaggs School of Pharmacy and Pharmaceutical Sciences, University of California San Diego, La Jolla, CA USA; 5 Present Address: Metaorganism Immunity Section, Laboratory of Immune System Biology, National Institute of Allergy and Infectious Diseases, National Institutes of Health, Bethesda, MD USA

**Keywords:** Cell division, Checkpoints

## Abstract

During early G1 phase, Rb is exclusively mono-phosphorylated by cyclin D:Cdk4/6, generating 14 different isoforms with specific binding patterns to E2Fs and other cellular protein targets. While mono-phosphorylated Rb is dispensable for early G1 phase progression, interfering with cyclin D:Cdk4/6 kinase activity prevents G1 phase progression, questioning the role of cyclin D:Cdk4/6 in Rb inactivation. To dissect the molecular functions of cyclin D:Cdk4/6 during cell cycle entry, we generated a single cell reporter for Cdk2 activation, RB inactivation and cell cycle entry by CRISPR/Cas9 tagging endogenous p27 with mCherry. Through single cell tracing of Cdk4i cells, we identified a time-sensitive early G1 phase specific Cdk4/6-dependent phosphorylation gradient that regulates cell cycle entry timing and resides between serum-sensing and cyclin E:Cdk2 activation. To reveal the substrate identity of the Cdk4/6 phosphorylation gradient, we performed whole proteomic and phospho-proteomic mass spectrometry, and identified 147 proteins and 82 phospho-peptides that significantly changed due to Cdk4 inhibition in early G1 phase. In summary, we identified novel (non-Rb) cyclin D:Cdk4/6 substrates that connects early G1 phase functions with cyclin E:Cdk2 activation and Rb inactivation by hyper-phosphorylation.

## Introduction

Excluding mitosis and cytokinesis, all other cell cycle phases, including the progression from early to late G1 phase, are morphologically difficult to distinguish. Hence, only a limited number of studies have successfully visualized cell cycle progression outside of mitosis in living cells^1,2^. Despite their important scientific contributions, most studies have relied on over-expression of reporter constructs, potentially interfering with the processes they are visualizing. The recent advances in gene editing and gene replacement technologies have now made it readily feasible to study and visualize signaling processes of endogenous genes at the single cell level^3,4^.

The gene product of the human CDKN1B locus (p27, Kip1, CDKN1B) binds with high affinity to cyclin A/E:Cdk2 complexes, rendering the complex kinase-inactive^5–7^. p27 is actively degraded in a phosphorylation-dependent and cell cycle-specific manner^8^. The kinase responsible for initiating p27 degradation is Cdk2 itself^9^, which generates a positive feedback loop that renders cell cycle progression unidirectional^10^. Genetic ablation of p27 induces a gene dosage-dependent increase of animal size, due to an increased number of total cells in all organs^11–13^. However, gross morphological abnormalities are absent making it feasible to use p27 as a sensor for Cdk2 activation.

We have previously demonstrated that cells entering the cell cycle select for active, mono-phosphorylated Rb by cyclin D:Cdk4/6 complexes that generate up to 14 different mono-phosphorylated isoforms that regulate Rb’s preferential binding to different E2F family members and induce a correct DNA damage response^14^. Moreover, Sanidas et al. both confirmed cyclin D:Cdk4/6 mono-phosphorylation, but also expanded these observations showing that mono-phosphorylation is a code for Rb to find cellular target proteins during early G1 phase^15^. At the end of early G1 phase, Rb becomes hyper-phosphorylated by activation of Cdk2 to trigger progression into late G1 phase, release of E2F transcription factors and subsequent entry into S phase^16^. However, if Cdk2 is artificially deleted, due to cellular plasticity, Cdk1 can substitute for Cdk2^16^. Interestingly, although mono-phosphorylated Rb is required to help mediate a correct DNA damage response, mono-phosphorylation of Rb is neither necessary nor required for successful cell cycle entry^14^, questioning the role of cyclin D:Cdk4/6 in Rb inactivation. In contrast, the continuous presence of cyclin D:Cdk4/6 kinase activity in early G1 phase is essential for cell cycle entry^17,18^, though the critical non-Rb substrate(s) remain unknown.

Here we used CRISPR/Cas9 gene editing and rAAV-mediated gene replacement to genetically tag endogenous p27 (Kip1) with mCherry in hTERT immortalized human retinal-pigmented epithelial cells (RPE1). Using this reporter for cell cycle entry, we performed single cell time-lapse microscopy and identified an essential cyclin D:Cdk4/6 phosphorylation gradient that is integrated with progression into late G1 phase. To reveal the identity of Cdk4/6 substrates, we performed whole proteomic and phospho-proteomic mass spectrometry and identified new early G1 phase cyclin D:Cdk4/6 substrates. Together, our findings demonstrate that G1 cell cycle progression proceeds by cyclin D:Cdk4/6 progressive phosphorylation of non-Rb substrates.

## Results

### Tagging endogenous p27 as a singe cell reporter for cell cycle entry

The use of fluorescent cell cycle reporter systems has greatly enhanced our understanding of cell cycle regulation^1,2^. However, the use of overexpressed reporters can biologically interfere with the very processes that they are intended to visualize. In contrast, endogenous proteins escape this obstacle, but are difficult to study on the single cell level. In an attempt to visualize late G1 phase entry in the most unperturbed way possible, we targeted the genomic locus of p27 in hTERT-RPE1 cells, which are immortalized human retinal pigmented epithelial cells, with a combination of CRISPR/Cas9 and rAAV to induce homology directed repair (HDR). We introduced mCherry-HA into the C-terminus of p27 (Supplemental Figure S1A)^19^. Integration- and zygocity-specific PCR revealed the presence of multiple recombined clones (Supplemental Figure S1B,C). To test the functionality of p27-mCherry-HA, we synchronized p27-mCherry-HA cells into G0 with serum-free media (SFM), G1-S phase with hydroxyurea (HU) and G2-M phase with nocodazole (Noco), then immunoprecipitated p27 with an anti-HA antibody. As expected, p27-mCherry-HA was fully functional and co-precipitated Cdk2, as well as cyclin E and A (Supplemental Figures S1D,E, S2). Moreover, consistent with endogenous p27, anti-HA p27-mCherry-HA immunoprecipitates preferentially bound to the active T160-phosphorylated form of Cdk2^20^.

DNA replication is directly coupled to histone transcription and translation. Hence, we complemented our p27-mCherry cell line by targeting the histone 2B (H2B) genomic locus with an enhanced green fluorescent protein (eGFP) (Supplemental Figure S3). To test the p27-mCherry-HA/H2B-eGFP cells for specific cell cycle profiles of eGFP and mCherry, we transfected these cells with siRNAs targeting Cdk2, Rb or control Luciferase (CTRL) for 48 h, followed by flow cytometry analysis (FACS). In CTRL siRNA treated cells, we identified three distinct populations of cells, p27-mCherry-high/H2B-eGFP-low, p27-mCherry-low/H2B-eGFP-low, and p27-mCherry-low/H2B-eGFP-high (Supplemental Figure S1F). Transfection of Cdk2 siRNA collapsed all populations into p27-mCherry-high/H2B-eGFP-low, demonstrating a strong G1 phase cell cycle arrest. In contrast, the Rb siRNA had the opposite effect and fostered the presence of p27-mCherry-low/H2B-eGFP-low/high populations, suggesting an accelerated cell cycle entry (Supplemental Figure S1F). These data prompted us to determine p27-mCherry and H2B-eGFP signals by time-lapse microscopy. We transfected H2B-eGFP/p27-mCherry cells with Cdk2, Rb and control siRNAs for 36 h, then started recording green and red fluorescence signals over the next 48 h. By aligning all H2B-eGFP signals to the point of cellular division/mitosis (M)^1^, we determined a strong cell cycle arrest and premature S phase entry phenotype for Cdk2 or Rb siRNA transfected cells, respectively (Supplemental Figure S1G). In line with these observations, Cdk2 depletion resulted in a dramatic increase in nuclear p27, while Rb depletion induced a strong reduction in nuclear p27 signals (Supplemental Figure S1H, Supplemental Videos 1–3). Taken together, these data demonstrate that endogenous p27-mCherry is fully functional and can serve as a read out for Cdk2 activation at the early to late G1 phase transition on the single cell level.

### Identification of a p27 inflection point

**Figure 1:**
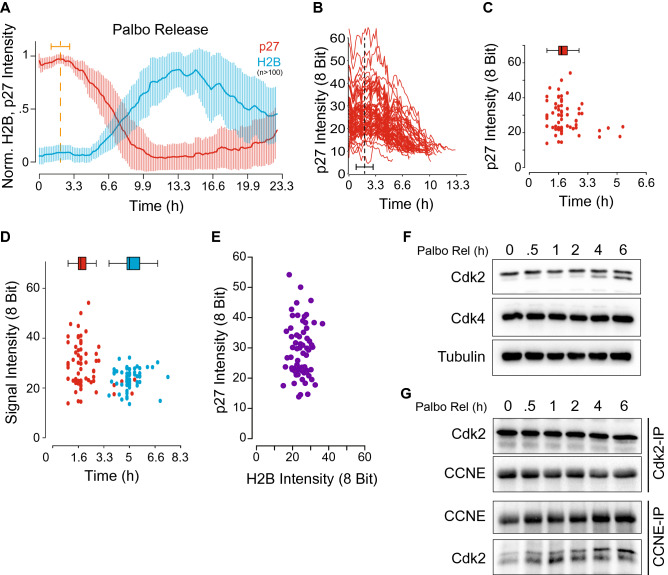
p27-mCherry, a single cell reporter for cell cycle entry. (**A**) Average over single cell p27-mCherry and H2B-eGFP signals during a Palbociclib release. Bold red and blue lines indicate an average over 10 for endogenous p27 or H2B, respectively. Thin vertical red and blue lines indicate the standard deviation at this time point over the past and future 5 time points. (**B**,**C**) Heterogeneity of single cell p27-mCherry signals. Data derived from (**A**). Note: the p27-mCherry inflection point is independent of a cells p27-mCherry level. Red dots: a cells p27-inflection point. (**D**,**E**) A cells p27 inflection point does not correlate with S phase entry (measured as 5% increase in H2B-eGFP signal). Red dots: p27-inflection point. Blue dots: S phase entry. Purple dots: single cell correlation between p27-inflection point and S phase entry. (**F**,**G**) Western blot and immune-precipitate of lysates, as cells treated in A. Note: Cdk2 activation becomes apparent between 2 and 4 h, whereas the complex between Cdk2 and cyclin E is preassembled.

To study the early to late G1 phase transition more comprehensively, we synchronized the p27/H2B reporter cells into early G1 phase by addition of the selective Cdk4/6 inhibitor Palbociclib (PD0332991, IBRANCE) for 48 h. After release from Palbociclib by extensive washing, we recorded single cell eGFP and mCherry signals by time-lapse microscopy over the next 22 h. After normalizing and aligning all single cell traces of eGFP and mCherry, we noted that the initial p27-mCherry intensity rose slightly before the presence of a sharp inflection point at ~ 1.8 h after Palbociclib washout (Fig. 1A, Supplemental Video 4). In contrast, H2B-eGFP signals started to increase only after 5.4 h of Palbociclib washout (Fig. 1A, Supplemental Video 4), suggesting a total length of late G1 phase in hTERT-RPE1 cells of ~ 3.6 h. Interestingly, although single cell p27 levels varied from cell to cell by a factor of 6, the timing of the inflection point remained unchanged in relationship to p27 levels (Fig. 1B,C). However, we were unable to identify a correlation between the p27 inflection point and the beginning of S phase (Fig. 1D,E). Since the loss of p27 protein directly correlates with the activation of Cdk2^21,22^, we analyzed the appearance of active Cdk2 by immunoblot. To do so, p27/H2B cells were arrested in early G1 phase with Palbociclib, released and harvested at indicated time points. We detected measurable active cyclin E:Cdk2 complexes as early as 2–4 h post-Palbociclib release (Fig. 1F,G, Supplemental Figure S2). Interestingly, cyclin E was stably complexed to Cdk2 during the entire time course (Fig. 1G). In contrast to a previous model where Cdk2 is activated by increasing levels of cyclin E protein, our data suggest that activation of cyclin E:Cdk2 at the early to late G1 phase transition is primarily dependent on T160 T-loop phospho-regulation.

### Cdk4/6 is dispensable after the p27 inflection point

**Figure 2:**
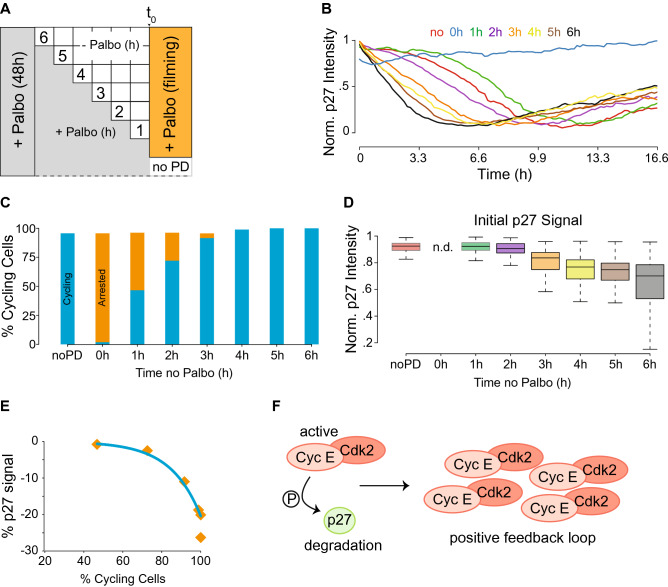
Cyclin D:Cdk4/6 is indispensable after the p27-mCherry inflection point. (**A**) Experimental scheme. In short: p27-mCherry cells were treated for 48 h with Palbociclib before Palbociclib was washed out for the indicated time periods. Before cells were subjected to time-lapse microscopy, fresh Palbociclib was added to all cells but a control sample. Cells were recorded for 20 h, their eGFP and mCherry signals computed to visualize the p27-inflection point. (**B**) An average p27-mCherry signal over 10 time-points for each treated sample is visualized. Note: a time-dependent p27-inflection point is demonstrated. (**C**) Bar graph, grouping all cells from A into cycling (blue) and not cycling or arrested (orange). Interestingly, cells release from 48 h of Palbociclib linearly. (**D**) Box plot visualizing the initial (first 3 time points) p27-mCherry signal intensity of single cells that committed to cellular division (5% increase in H2B-eGFP signal) and its change over time. (**E**) The percentage of cycling cells per time point of no Palbociclib is negative exponentially correlated to the drop in initial p27-mCherry signal. (**F**) Model: The phosphorylation of a relatively small number of p27 proteins is sufficient to induce a positive feedback loop leading to irreversible Cdk2 activation and cell cycle commitment (adapted from ref.^8^).

Cyclin D:Cdk4/6 activity is required for late G1 phase entry, whereas Rb mono-phosphorylation is not required^14^. To ascertain when cyclin D:Cdk4/6 activity becomes dispensable for cell cycle entry, we synchronized p27/H2B cells with Palbociclib in early G1 phase, then individual wells were washed and released from Palbociclib for 1 to 6 h before Palbociclib was added back, followed by recording p27-mCherry and H2B-eGFP signals by time-lapse microscopy (Fig. 2A). After normalizing single cell tracing, we identified a time-dependent window between 1 and 2 h of Palbociclib release at which point the activity of cyclin D:Cdk4/6 became dispensable for cells to enter late G1 phase and progress into S phase (Fig. 2B). During the course of this experiment, we detected the presence of two distinct populations of cells. The first population entered late G1 phase (cycling), while the second population did not and remained arrested. The relative size of the arrested cell population correlated linearly with the amount of Palbociclib-free time cells experienced before Palbociclib was added back and fluorescence recording was initiated (Fig. 2C). By looking at the cycling cell population more closely, we were able to determine the initial p27 value for all single cycling cells. This reveled a sharp decrease in the initial p27 signal after cells had been released from Palbociclib for more than 2 h (Fig. 2D). This is entirely consistent with our previous observation that cells experience a p27 inflection point after ~ 1.8 h of Palbociclib release. Moreover, the percentage of cycling cells correlated exponentially with the initial drop in p27 single cell signal (Fig. 2E). These data demonstrate that endogenous p27 is most sensitive to Cdk2 activation during the early to late G1 phase transition. Furthermore, our data support the current model in which few active cyclin E:Cdk2 complexes phosphorylate and inactivate many p27 molecules, initiating a positive feedback loop that induces full cyclin E:Cdk2 activation (Fig. 2F).

### The p27 inflection point links serum sensing and Cdk2 activation

Having identified a p27-inflection point, we questioned its timely localization during early G1 phase progression with respect to serum sensing and Cdk2 activation. Therefore, we synchronized cells in G1 phase with Palbociclib, then released for varying time periods before serum was withdrawn and cells were subjected to time-lapse video microscopy (Supplemental Figure S3A). We found that all cells entered the cell cycle independent of the time without Palbociclib, as determined by the presence of a p27-inflection point (Supplemental Figure S3B). Cell cycle commitment was further confirmed by determining total cell numbers and nuclear size during the release experiment. As expected, cells under all tested conditions performed cellular division and underwent nuclear growth with the expected delays due to the different Palbociclib release time points (Supplemental Figures S3A and S3C,D).

Cdk2 activity is required for cell cycle entry, thus, we synchronized cells with Palbociclib, released them for different periods of time before they were exposed to Roscovitine to block Cdk2 activity and analyze the p27-inflection point (Supplemental Figure S3E). When compared to control treated cells, only cells that were released from Palbociclib for more than 2 h displayed p27-degradation kinetics that were similar to the previously observed kinetics (Supplemental Figure S3F). Cells that had less than 2 h of Palbociclib free time prior to Roscovitine addition displayed no to severely delayed p27-degradation kinetics (Supplemental Figure S3F). As expected, mitotic division was blocked in all cells exposed to Roscovitine (Supplemental Figure S3G), due to the fact that Cdk2 activity is required for S phase progression and DNA replication (Supplemental Figure S3H). Thus we concluded that the Palbociclib-dependent cell cycle arrest happens between serum sensing and Cdk2 activation and that the p27-inflection point represents most likely what has been described as the restriction point.

### Identification of a Cdk4/6-dependent phosphorylation gradient

**Figure 3:**
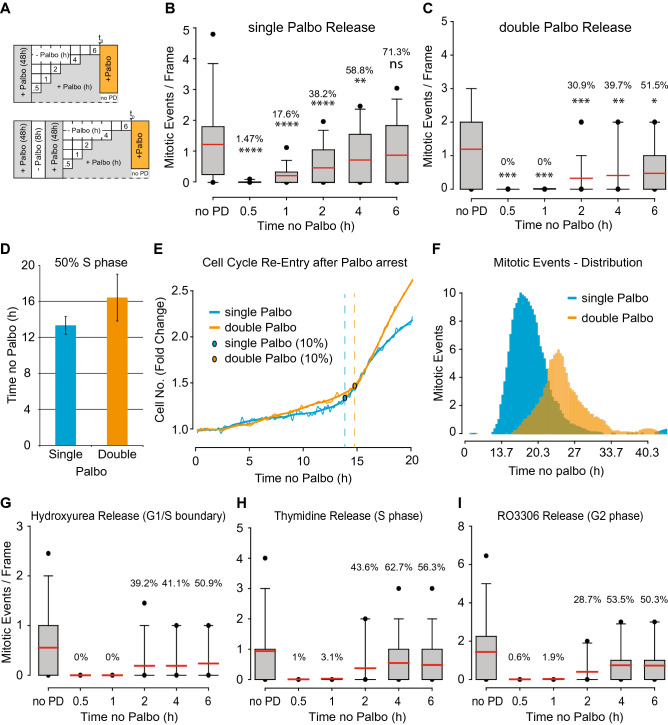
Identification of a cyclin D-dependent phosphorylation gradient during early G1 phase. (**A**) Scheme visualizing the experimental set up of this figure. In short: cells were treated with Palbociclib for the indicated period of time, released and subjected to time-lapse microcopy. (**B**,**C**) Cells treated as depicted in (**A**) were analyzed for their ability to undergo cellular division. Red bar: average of population, Percentage: percent of cells per time point that undergo cellular division, Asterisks (based on Mann–Whitney-U-Test): ns: *p* > 0.05, *: *p* ≤ 0.05, **: *p* ≤ 0.01, ***: *p* ≤ 0.001, ****: *p* ≤ 0.0001. (**D**–**F**) H2B-eGFP signal of single cell traces of cells from B and C were analyzed for S phase entry (**D**–**E**) and mitotic division (**F**). Turning points of single cells were set as an increase of 10% in H2B-eGFP signal over 5 time-points (1 h). To (**D**) Bar graph, visualizing the average over all determined single cell turning points of B and C. To (**E**) H2B-eGFP single cell traces of cells from B and C. Blue and orange circles: turning points. To (**F**) Distribution of mitotic divisions from cells in B and C. (**G**–**I**) Cells release from a single Palbociclib arrest in a step-wise manner if previously synchronized outside of G1 phase. Percentage: percent of cells per time point that undergo cellular division.

During the above-described experiments, we observed that Palbociclib released cells entered the cell cycle in a linear fashion (Figs. 2C, 3B–D). This was particularly surprising as we expected Palbociclib to arrest all cells at a distinct position at the start of early G1 phase as opposed to throughout early G1 phase. To rule out a p27-mCherry artifact, we independently verified results from Fig. 2 and 3, but evaluated cell cycle entry purely by a cell’s ability to perform mitotic division at the end of each cycle (Fig. 3A). Cells released from Palbociclib after a 48 h arrest entered the cell cycle in a linear fashion (Fig. 3B). This observation supported the notion that Cdk4/6 deficiency arrests cells throughout early G1 phase. To test this hypothesis, we double arrested cells by first treating with Palbociclib for 48 h, followed by a wash out and release for 8 h, and then adding back Palbociclib for an additional 48 h before the cells were released again and scored for their ability to perform cellular division (Fig. 3A). In sharp contrast to the single Palbociclib arrested cells, double Palbociclib arrested cells displayed a step-wise cell cycle entry pattern (Fig. 3C) that required more time to enter S and M phases (Fig. 3D–F). Importantly, this step-wise behavior occurred regardless of whether the first arrest was initiated at S phase (by hydroxyurea or thymidine addition) or G2/M phase (by RO3306 addition) (Fig. 3G–I), suggesting that Cdk4/6 activity is required to phosphorylate an as yet to be identified early G1 phase substrate(s) until a critical threshold is reached that results in activation of cyclin E:Cdk2 by CAK phosphorylation of T160 in the T-loop.

**Figure 4:**
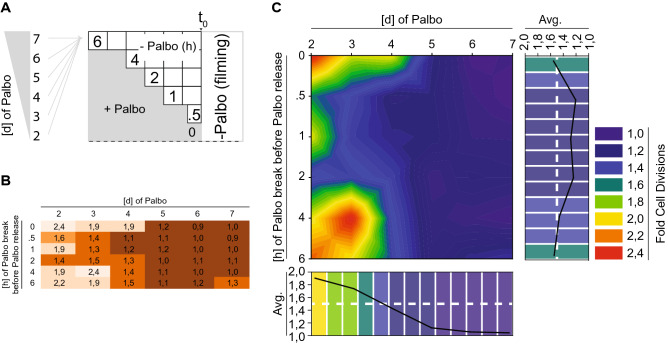
The Cdk4/6 phosphorylation gradient is time-sensitive. (**A**) Scheme visualizing the experimental set up of this figure. In short: cell were treated with Palbociclib for the indicated period of time (left), released for the indicated period of time (right) and subjected to time-lapse microcopy. (**B**,**C**) Single cells were traced and analyzed by their ability to undergo mitotic division. To (**B**) Normalized data (Fold Cell Division): 1 = no division, 2 = 1 division. To (**C**) Heat map of data from B. Color code represents an average cells ability to undergo mitotic division after being treated as in (**A**).

The presence of a Cdk4/6-dependent phosphorylation gradient raised the question of its stability over time. Therefore, we set up an experiment in which the duration of Palbociclib treatment was titrated against Palbociclib-free time before cells were recorded by time-lapse microscopy for 24 h (Fig. 4A). As previously observed, cells treated with Palbociclib for 48 h and released, displayed a linear release behavior and underwent cellular division by a factor of ~ 2 (Fig. 4B,C). However, the fold of cellular division was dramatically reduced from 1.8 to 1.4 after 3 and 4 days of Palbociclib treatment and release, respectively (Fig. 4B,C). Moreover, the linear release behavior was essentially diminished by all Palbociclib treatments of 4 days or longer (Fig. 4B,C). Most surprisingly, cells treated longer than 4 days not only had lost their linear release profile, but also displayed severe problems in re-entering the cell cycle, with 5 to 7 days containing < 20% of cells entering the cell cycle (Fig. 4B,C). Taken together, these data demonstrated that the Cdk4/6-dependent phosphorylation gradient is time-sensitive, but lost after Palbociclib treatments exceeding 3 days. Thus, our data points to the requirement of basal Cdk4/6 activity during a prolonged early G1 phase arrest to prevent permanent cell cycle arrest and potentially senescence.

### Proteomic signature of early G1 Palbociclib-treated cells

**Figure 5:**
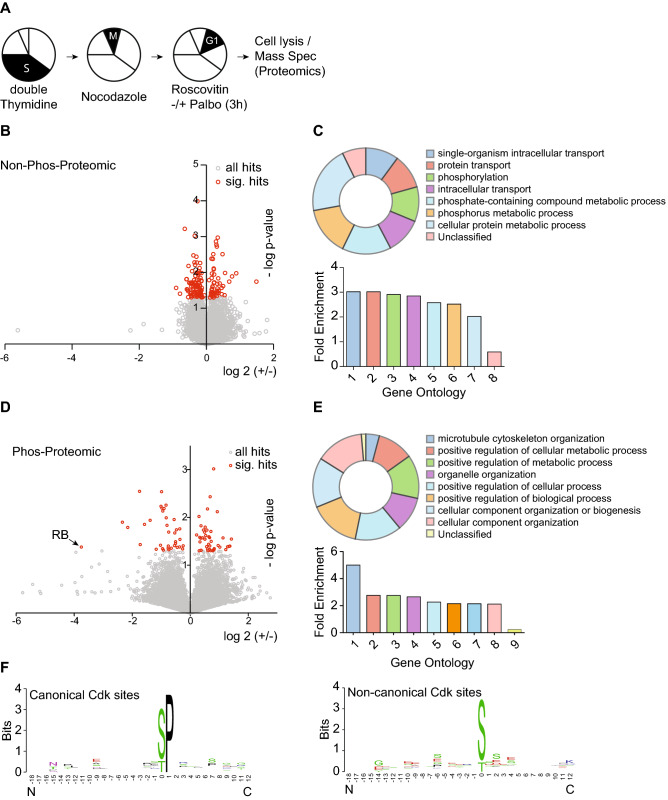
Proteomic- and phosphor-proteomic signature of early G1 cells treated with Palbociclib. (**A**) Scheme visualizing the experimental set up of this figure. In short: cells were S phase arrested by double Thymidine blockage, released into Nocodazole to induce pro-metaphase blockage in mitosis, harvested by mitotic shake-off and subsequently released and re-plated for 1 h before Roscovitine [15 μM] and either DMSO or Palbociclib was added to the cells for 3 h. (**B**) Scatter plot of proteins identified by whole proteomic mass-spectrometry. Note: Proteins undergoing a significant regulation are displayed in red. (**C**) Significant Hits from B were used for Gene-Ontology analysis. Significantly enriched pathways are displayed as their overall contribution (top panel) and their fold enrichment over background (bottom panel). (**D**) Scatter plot of phosphor-peptides identified by mass-spectrometry. Note: Peptides undergoing a significant regulation are displayed in red. (**E**) Significant Hits from D were used for Gene-Ontology analysis. Significantly enriched pathways are displayed as their overall contribution (top panel) and their fold enrichment over background (bottom panel). (**F**) All significant hits from D were used for canonical and non-canonical Cdk consensus site motif identification.

The most widely studied Cdk4/6 substrate is the retinoblastoma protein (Rb) along with two related proteins, p107 (RbL2) and p130 (RbL3). However, our previous data demonstrated that cyclin D:Cdk4/6 complexes exclusively mono-phosphorylates Rb throughout early G1 phase with no multi-phosphorylation observed until Cdk2 hyper-phosphorylation^14^, suggesting that the time-sensitive gradient that we identified here was independent of Rb. To identify new early G1 phase cyclin D:Cdk4/6 substrates, we synchronized hTERT-RPE1 cells in S phase with a single dose of 2 mM Thymidine for 24 h, followed by PBS-washout and release into fresh media for 14 h in the presence of Nocodazole to induce a G2/M phase arrest. G2/M phase cells were then harvested by mitotic-shake off, PBS washed and released into fresh media for 1 h prior to treatment with Roscovitine or Roscovitine plus Palbociclib (Fig. 5A). After 3 h of Roscovitine or Roscovitine plus Palbociclib treatment, cells were harvested, lysed and processed for whole proteomic and phospho-proteomic analysis^23^. Using a 3 × 3 × 3 biological triplicate format, we identified a total of 23,009 phospho-peptides, correlating to 7,537 independent proteins with 147 proteins displaying significant positive or negative change in abundance (Fig. 5B). Gene ontology analysis revealed transport, phosphorylation and metabolic processes as the most significant enriched biological processes amongst the significant hits (Fig. 5C). With respect to the phospho-peptides, only 82 peptides of the 23009 identified showed a significant positive or negative change in response to Palbociclib (Fig. 5D). Gene ontology analysis identified cytoskeleton and metabolic processes as the most enriched genes (Fig. 5E). We further increased the stringency by the presence of phospho-Sp/Tp for canonical Cdk motifs and identified 24 canonical Cdk sites phospho-peptides, of which 13 were up regulated and 11 were down regulated. Phospho-peptide motif analysis identified the proline residue in the plus 1 position as the only enriched amino acid, confirming the S/Tp dependency of cyclin-dependent kinases and suggesting it to be the only stringent determinant (Fig. 5F, Supplemental Figure S4). Based on our analysis, we propose that one or multiple of our newly identified Palbociclib-sensitive, Cdk4/6 phospho-proteins are directly involved in regulating the Cdk4/6-dependent phospho-gradient that controls early G1 phase progression in single cells.

## Discussion

### An Rb independent phosphorylation-gradient during early G1 phase

Rb is a cyclin D:Cdk4/6 substrate with 14 phospho-acceptor sites^14^, and artificial phosphorylation of Rb in a test tube with cyclin D:Cdk4/6 results in phosphorylation at all sites, generating hyper-phosphorylated Rb. Furthermore, Rb depletion and/or artificial forced hyper-phosphorylation resulted in premature cell cycle entry, hence, a model that cyclin D:Cdk4/6 progressively multi-phosphorylates Rb, thereby negatively regulating its transcriptional repressor activity, appeared plausible. In line with this, genetic ablation or chemical interference of cyclin D:Cdk4/6 kinase activity results in early G1 cell cycle arrest, emphasizing the importance of cyclin D:Cdk4/6 kinase activity for early G1 phase progression.

In direct contrast to this model, we and others have previously demonstrated that cyclin D:Cdk4/6 does not multi-phosphorylate Rb in cells, but strictly mono-phosphorylate Rb on 14 phosphor-acceptor sites, thereby regulating Rb’s binding to the E2F family of transcription factors and a multitude of cellular targets^14,15^. At the early to late G1 transition, Cdk2 performs the inactivating Rb hyper-phosphorylation to release E2F transcription factors, whereas if Cdk2 is artificially deleted, due to cellular plasticity, Cdk1 will substitute for Cdk2^16^. Importantly, while Rb mono-phosphorylation is crucial for a DNA damage response, it is not essential for early G1 phase progression, suggesting additional, yet unknown functions and targets of cyclin D:Cdk4/6 during early G1 phase. We note, however, that entire cell's compliment of Rb is strictly mono-phosphorylated during early G1 phase in all normal and tumor cell types assayed (there is no naturally occurring un-phosphorylated Rb in early G1 phase or any multi-phosphorylated Rb).

Here we used CRISPR/Cas9 gene editing and rAAV gene replacement to generate a single cell reporter for the early to late G1 phase transition. Our p27-mCherry reporter leads us to several important conclusions: (1) single cell p27 levels are heterogeneous and independent of cell cycle entry, (2) single cells build up a cyclin D:Cdk4/6-dependent phosphorylation gradient that regulates cell cycle entry, and (3) the phosphorylation gradient is time-sensitive.

p27 is a bona fide cyclin A/E:Cdk2 inhibitor, whose Cdk2 phosphorylation- and Skp2-mediated degradation induces a positive feedback loop to ensure the irreversibility of cell cycle entry. Over-expression of wild-type p27 blocks cells in early G1 phase with active cyclin D:Cdk4/6 and mono-phosphorylated Rb, demonstrating that an excess of p27 is not tolerated for G1 progression. Our data find the single cell level of p27 to be very heterogeneous with cellular differences of up to sixfold. Nevertheless and irrespectively of the p27 level, all cells entered the cell cycle with similar kinetics, suggesting the presence of a modulating mechanism that orchestrates p27 levels with cell cycle entry timing. Due to the fact that exogenous p27 over-expression blocks cells from S phase entry, we favor the presence of a single cell p27 level threshold, that when reached by intrinsic or external factors prevents cells from cell cycle entry and may lead to permanent cell cycle exit.

Rb is exclusively mono-phosphorylated during all of early G1 phase and only hyper-phosphorylated when Cdk2 activity becomes detectable. Hence, we argue that the cyclin D:Cdk4/6 phospho-gradient is Rb-independent. Other cyclin D:Cdk4/6 substrates have been described, among them the FOXM1 transcription factor^24^. Interestingly, FoxM1 has been characterized to regulate critical G1/S phase genes with multi- or hyper-phosphorylated FOXM1 being the most active^24^. Despite its proposed function, we failed to identify any FOXM1 phosphor-peptides in our proteomic screen, probably due to low FOXM1 abundance. However, FOXM1 is most likely not the only critical Cdk4/6 substrate. If a FOXM1 mutant, containing phosphor-mimetic residues at all 15 predicted phosphor-acceptor sites, contributes to Palbociclib resistance remains to be seen. However, we wish to emphasize that the textbook model that a cyclin D:Cdk4/6 substrate is progressively phosphorylated until a cell cycle-entering threshold is reached is factually incorrect. In fact, we believe that our previous and present data support this model; however, we conclude that Rb is not the critical progressive phosphorylation substrate of cyclin D:Cdk4/6 during early G1 phase.

### Proteomic profiling of Palbociclib targets

While Rb is clearly the most well studied cyclin D:Cdk4/6 substrate, there has been a paucity of cyclin D:Cdk4/6 substrate. However, up until recently, a comprehensive mass spectrometry approach has not been possible due to the lack of specific reagents. The development of Palbociclib has closed this gap. Hence, we performed whole proteome and phosphor-proteome mass spectrometry and compared early G1 phase cells treated either with DMSO or Palbociclib for 3 h. Our approach identified new cyclin D:Cdk4/6 substrates, as well as proteins that experience abundance changes in response to Palbociclib. Among them, we identified 11 canonical Cdk phosphor-acceptor sites that displayed significant down regulation. Gene ontology (GO) analysis revealed a significant increase of genes involved in metabolism. This goes well with the fact that Cdk4 has been linked to several aspects of metabolism, including glucose metabolism and mitochondrial biogenesis^25,26^. With respect to the identified Cdk4/6-dependent phosphorylation-gradient, we propose its mechanistic linkage may be relevant to one or more of our identified phosphor-proteins. However, at this point in time, we do not yet know the identify of the critical cyclin D:Cdk4/6 targets.

## Materials and methods

### Cell culture and synchronization

Human hTERT-RPE1 cells were cultured at 37 °C, in 5% CO2 atmosphere and DME/F12 media (Life Technologies), supplemented with 10% heat-inactivated fetal bovine serum (FBS) and penicillin/streptomycin (100 μg/ml). For synchronizations, cells were treated with 2 mM thymidine (Sigma-Aldrich), 4 mM hydroxy urea (Sigma-Aldrich), 9 μM RO3306 (Sigma-Aldrich), 15 μM Roscovitine (Sigma-Aldrich) for 24 h prior to release, or with 1 μM Palbociclib (PD-0332991, Ibrance; Selleck Chemicals) for 48 h if not indicated otherwise. Cells were released by washing four times with full media and subsequently analyzed or treated as indicated.

### CRISPR and rAAV cloning procedures

A p27-specific gRNA (5′-GTCAAACGTAAA CAGCTCGGT-3′) was designed and cloned into pX330 using previously published online algorithms and protocols, respectively^3,27^. For cloning of human p27 left and right homology arms (LHA, RHA) the following DNA oligonucleotides were used: p27-LHA-f: 5′-GGCGGTCGTGCAGACCCGGGAGAAAGATGTC-3′, p27-LHA-r: 5′-CGTTTGACGT CTTCTGAGGCCAGGCTTCTTGG-3′, p27-RHA-f: 5′-GATCACTAAAGGAGCACGCACT GGAACCCGGGGCC-3′, p27-RHA-r: 5′-CGGCACAATTAGCATACACTGGCTGACGTGTTCCT GGATTGC-3′. Homology arms were amplified from genomic DNA of hTERT-RPE1 cells and cloned into pAAV-SEPT, flanking a C-terminal mCherry-2A-Neomycin tagging and selection cassette^19^. Infectious rAAV particles were generated as previously described^3^.

### Time-lapse video microscopy, data analysis

For cell cycle analysis, hTERT-RPE1 cells were seeded into either 6-well or 96-well chambers, treated as indicated and imaged using a Nikon ECLIPSE Ti microscope equipped with a CoolLED pE-1 excitation system and a × 20/0.75 air Plan Apo objective (Nikon). For single cell tracing, hTERT-RPE1 cells were seeded into 96-well chambers, treated as indicated and imaged using the high-throughput imaging systems Yokogawa CQ1 or Yokogawa CV7000S (Yokogawa Electric Corporation). Images were acquired by simultaneously recording of 2 × 3 or 3 × 3 positions every 10 or 20 min and automatically stitched and processed by Fiji (version 2.0.0-rc-43/1.50e)^28^.

### Single cell tracing

Images were imported to Fiji and stack converted to binary files with the ‘Li’ method and ‘Dark’ background and through calculation of thresholds for each image. Subsequently, the TrackMate plugin (v2.8.1)^29^ was used for single cell tracing with the following settings: DoG detector, 20 μm blob diameter with a threshold of 0.5, 15 μm of linking distance, 15 μm of gap-closing maximal distance and 2 frames for maximal gap-closing. Single cell traces were exported as csv-files for further analysis.

### Mass spectrometry

#### Cell lysis, protein digestion, phosphor-enrichment and TMT labeling

Cells were lysed in a buffer composed of 3% SDS, 75 mM NaCl, 1 mM NaF, 1 mM beta-glycerophosphate, 1 mM sodium orthovanadate, 10 mM sodium pyrophosphate, 1 mM PMSF and 1X Roche Complete mini EDTA free protease inhibitors in 50 mM HEPES, pH 8.5^30^. Lysates were passed through a 21 gauge needle 20 times and sonicated for 5 min to ensure complete lysis. Cellular debris was pelleted via centrifugation at 14,000 RPM for 5 min, and resultant supernatants were used for processing.

Protein disulfides were reduced with DTT and alkylated with iodoacetamide as previously described^31^. Reduced/alkylated proteins were precipitated by methanol-chloroform precipitation^32^. Precipitated proteins were re-suspended in 1 M urea, 50 mM HEPES, pH 8.5 for proteolytic digestion. Digestion was performed in a two-step process; proteins were first digested with LysC overnight at room temperature, then with trypsin for 6 h at 37 °C. Digestion was quenched by adding TFA, and peptides were desalted with C_18_ solid-phase extraction columns as previously described^33^. Eluate peptide concentrations were determined by BCA assay, and peptides aliquoted into 2 mg portions, which were lyophilized and stored at − 80 °C until phosphor-peptide enrichment.

Phosphor-peptides were enriched with an immobilized metal affinity chromatography (IMAC) approach as previously described^34,35^. Briefly, 8 mg of titanium dioxide beads was used per 2 mg of peptide starting material. Enriched peptides were desalted with solid-phase extraction columns as described above, then lyophilized and stored at − 80 °C until they could be labeled for quantitation.

Phosphor-peptides were labeled with 10-plex tandem mass tag (TMT) reagents^36,37^ as previously described^38^. TMT reagents, at a concentration of 20 μg/μL in dry acetonitrile were used for labeling. Lyophilized peptides were re-suspended in 30% acetonitrile in 200 mM HEPES, pH 8.5 and 5 μL of the appropriate TMT reagent will be added to the sample. The labeling was conducted for 1 h at room temperature, and then quenched by the addition of 6 μL of 5% hydroxylamine, which was allowed to react for 15 min at room temperature. Labeled samples were then acidified by adding 50 μL of 1% trifluoroacetic acid. Differentially labeled samples were pooled into one multiplex experiment and then desalted via solid-phase extraction as described above, then lyophilized and stored at − 80 °C until fractionation.

#### Basic pH reverse-phase fractionation

Samples were fractionated by basic pH reverse-phase liquid chromatography^39^. Lyophilized samples were re-suspended in 5% formic acid/5% acetonitrile and separated on a 4.6 mm × 250 mm C_18_ column on an Ultimate 3000 HPLC with a fraction collector, degasser and variable wavelength detector. Separation was performed over a linear gradient of 8 to 28% acetonitrile in 10 mM ammonium bicarbonate in 60 min with a flow rate of 0.5 mL/min. In total, 96 fractions were collected and combined in a concatenated manner as previously described^33^. Resultant fractions were lyophilized and re-suspended in 5% formic acid/5% acetonitrile for identification and quantification by LC-MS2/MS3.

#### Liquid chromatography mass spectrometry

All aspects of mass spectrometry experiments performed here were used the same text as previously published^40^. All LC-MS2/MS3 experiments were performed on an Orbitrap Fusion mass spectrometer with an in-line Easy-nLC 1000 with chilled autosampler. Peptides were separated on a home pulled, home packed column (inner diameter, 100 μm; outer diameter, 360 μm). The column was packed first with approximately 0.5 cm of C_4_ resin (5 μm, 100 Å) followed by approximately 0.5 cm of C_18_ resin (3 μm, 200 Å) and then to a final length of 30 cm with C_18_ (1.8 μm, 120 Å). Peptides were eluted with a linear gradient from 11 to 30% acetonitrile in 0.125% formic acid over 165 min at a flow rate of 300 nL/minute and heating the column to 60 °C. Electrospray ionization was achieved by applying 2000 V at the inlet of the column.

All aspects of mass spectrometry experiments performed here were used the same text as previously published^40^. The mass spectrometer was operated in data-dependent mode, with a survey scan performed over a mass to charge (m/z) range of 500–1200 at a resolution of 120,000 in the Orbitrap. For the MS1 survey scan, automatic gain control (AGC) was set to 5 × 10^5^ with a maximum injection time of 100 ms and the s-lens set to an RF of 60. The most abundant ions observed in the survey scan were subjected to MS2 and MS3 analysis for identification and quantitation respectively. For this analysis, Top Speed mode was utilized, enabling the instrument to acquire a maximum number of spectra in a 5 s experimental cycle. Data collected at MS1, MS2 and MS3 levels were centroided.

All aspects of mass spectrometry experiments performed here were used the same text as previously published^40^. For MS2 analysis, ions above an intensity threshold of 5 × 10^4^ were selected for fragmentation. They were isolated in the quadrupole portion of the mass spectrometer with an isolation window of 0.5 m/z, and then fragmented with collision-induced dissociation with a normalized energy of 30%. Fragment ions are detected with rapid scan rate setting enabled in the ion trap, with an AGC setting of 1 × 10^4^ and maximum injection time of 35 ms.

All aspects of mass spectrometry experiments performed here were used the same text as previously published^40^. MS3 analysis was performed using the synchronous precursor selection setting to maximize sensitivity for quantification of TMT reporter ions^36^. A maximum of 3 MS2 ions are simultaneously isolated and fragmented for MS3 analysis. An isolation window of 2 m/z was utilized to isolate MS2 fragments, and these were fragmented further with high energy collision dissociation at a normalized energy of 50%. Fragment ions were detected in the Orbitrap at a resolution of 60,000 with a low mass of 110 m/z for MS3 analysis. The MS3 AGC was set to 5 × 10^4^ with a maximum ion injection time of 150 ms. MS2 ions in the range of 40 m/z below and 15 m/z above the precursor (MS1) were excluded from selection for MS3.

#### Data processing and analysis

Data was processed using the ProteomeDiscoverer 2.1 software package. Sequest was used to assign identities to MS2 spectra searching against the Uniprot database of Human entries^41^. The database was appended to include a decoy database comprised of all protein sequences in reversed order for downstream false discovery estimation^42,43^. Search parameters included a 50 ppm MS1 mass tolerance, static modifications of 10-plex TMT tags on lysines and peptide n-termini and carbamidomethylation of cysteines. Variable modifications included oxidation of methionines and phosphorylation of serine, threonine and tyrosine residues. Data were filtered to a peptide and protein false discovery rate of less than 1% using the target-decoy search strategy^44^. Peptides matching to multiple proteins were assigned to the protein containing the largest number of matched redundant peptides following the law of parsimony^34^.

TMT reporter ion intensities were extracted from MS3 spectra for quantitative analysis. Spectra used for quantitation had to meet the requirements of greater than 10 average signal to noise per label and isolation interference of less than 25%^38^. Data were normalized in a multi-step process, whereby they are first normalized to the average for each peptide, and then to the median of all averages. To account for slight differences in amounts of protein labeled, these values were then normalized to the median of the entire dataset and reported as final normalized signal to noise ratios per peptide per sample.

#### Bioinformatics and biostatistics

Movies from microscope were processed and exported as xls files separately for p27 and H2B measures. Files were merged by using a 1100 pixel radius around every H2B data point for every cell. For all H2B data points without a close p27 data point, p27 was set to zero. p27 data points without H2B data points in close vicinity were ignored. All cells without time point zero were filtered out, to avoid complicated alignment algorithms. To normalize for measurement inaccuracy and to fill missing data points each data point for H2B and p27 was smoothed by the median within a +/− 5 time point windows. Next, all cells with a measured time of less than 10 h (60 time points) and all cells with no p27 signal were filtered out. Finally the distribution of H2B and p27 over time per cell was scaled down to values between 0 and 1. All plots show the data smoothed with the median and normalized to 1. To show graphs about trends in the population, all p27 and H2B values for all cells at every specific time point were averaged, using the mean. For defining a cell as cycling an increase of 60% between the lowest H2B and the highest H2B value was required. Cells with no H2B signal increase or less than 60% were defined as not cycling. To calculate the inflection point, the maximum peak in the p27 data was found for every cell. To ensure the global maxima, the 10 time points before the restriction point were required to increase, whereas the 10 time points after the restriction point were required to decrease in signal. To calculate the turning point for the H2B signal the global minima was calculated, requiring the last 10 time points to decrease and the next 10 time points to decrease in signal.

#### Transient transfection

For siRNA transfections, 1 μL of a 100 μM siRNA stock solution was mixed with Lipofectamine RNAiMAX Transfection Reagent (Thermo Fisher Scientific Inc.) and Optimem (Gibco) in a ratio of 1:1:100 (100 nM:1 μL:100 μL). siCdk2 and siRB and control obtained from Thermo Fisher Scientific Inc. For plasmid DNA transfections, 2 μg of plasmid DNA was mixed with Lipofectamine 2000 Transfection Reagent (Thermo Fisher Scientific Inc.) and Optimem (Gibco) in a ratio of 1:3:100 (1 μg:3 μL:100 μL). Both mixtures were incubated at room temperature for 30 min and subsequently added drop-wise to cells. While siRNA transfections were performed in the presence of full media, plasmid DNA transfections were done in the presence of serum-free media for 4 h after which full media was added back.

#### Cell extracts, immuno-precipitation and blotting

Preparation of lysates and immunoblot analyses were performed as described previously^45^ using Tris lysis buffer (50 mM Tris–HCl pH 7.8, 150 mM NaCl, 1% IGEPAL CA-630) containing 100 mM NaF, 100 mM β-glycerophosphate, 20 μg/ml RNase A, 20 μg/ml DNase and 1/300 protease inhibitor cocktail (P8340, Sigma–Aldrich) and 1/100 phosphatase inhibitor cocktail #2 (P5726, Sigma–Aldrich). Antibodies used in this study were purchased from the following sources: rabbit anti-Cdk2 (M2 SC-163, Santa Cruz Biotechnology), mouse anti-tubulin (Developmental Studies Hybridoma Bank, University of Iowa), mouse anti-Cyclin A (SC-751, Santa Cruz Biotechnology), mouse anti-cyclin E (SC-198, Santa Cruz Biotechnology), rabbit anti-CDK4 (sc-260, Santa Cruz Biotechnology), mouse anti-Rb (G3-245, BD Pharmingen), rabbit anti-p27 (sc-528, Santa Cruz Biotechnology) and anti-HA High affinity (3F10, 11867423001, Sigma-Aldrich). Secondary antibodies used for western blot analysis were goat anti-mouse (31430, Thermo Scientific) and, goat anti-rabbit (31460, Thermo Scientific). Mouse anti-tubulin hybridoma cell line (clone #12G10) was developed by J. Frankel and E.M. Nelson under the auspices of the NICHD and maintained by the Developmental Studies Hybridoma Bank.

## Supplementary Information

Supplementary Information 1.

Supplementary Video 1.

Supplementary Video 2.

Supplementary Video 3.

Supplementary Video 4.

Supplementary Information 2.
