# Supplementary material for: A Cdk4/6-dependent phosphorylation gradient regulates the early to late G1 phase transition
Source: Sci Rep. 2021 Jul 19;11:14736. doi: 10.1038/s41598-021-94200-w (PMC8290049; doi:10.1038/s41598-021-94200-w)

Supplemental Figures

A Cdk4/6-Dependent Phosphorylation Gradient Regulates the Early to Late G1 Phase Transition.

Manuel Kaulich[1,2,*], Verena M Link[1,5], John D Jr Lapek[3,4], Yeon J Lee[1], Christopher K Glass[1], David J Gonzalez[3,4], and Steven F Dowdy[1,*]

[1] Department of Cellular and Molecular Medicine, University of California San Diego, La Jolla, CA, USA
[2] Institute of Biochemistry II, Goethe University, Frankfurt am Main, Germany
[3] Department of Pharmacology, University of California San Diego, La Jolla, USA,
[4] Skaggs School of Pharmacy and Pharmaceutical Sciences, University of California San Diego, La Jolla, CA, USA
[5] Current address: Metaorganism Immunity Section, Laboratory of Immune System Biology, National Institute of Allergy and Infectious Diseases, National Institutes of Health, Bethesda, MD, USA.

*Correspondence should be addressed to: Steven Dowdy, sdowdy@ucsd.edu or Manuel Kaulich, kaulich@em.uni-frankfurt.de

Supplemental Figures

Figure S1. Generation of hTERT-RPE1_H2B-eGFP_p27-mCherry.
(**A**) Graphic illustrating the homology template (HDR template). (**B**) Site and integration-specific PCR to identify faithfully recombined p27-mCherry clones. (**C**) PCR on faithfully recombined clones from (B), to distinguish between hetero- and homozygous clones. Positive PCR amplification indicates the presence of a heterozygous clone. (**D**) A single clone from (C) was amplified and either analyzed as a asynchronous (AS) or synchronized with serum-free media (SFM), Hydroxy-urea (HU), or Nocodazole. Lysates were subjected to western blot. (**E**) Lysates from (D) were subjected to HA-IP and blotted for the co-immunoprecipitation of Cdk2, cyclin A and E. (**F**) Time-lapse video analysis of parental RPE1, RPE1_H2B-eGFP and RPE1_H2B-eGFP_p27-mCherry cells. No line displays significant changes in the overall cell cycle timing. (**G**) p27-mCherry cells were either transfected with CTRL, Cdk2 or Rb siRNAs for 48 h and subsequently analyzed by FACS to their content of eGFP and mCherry. Note: three distinct subpopulations can be identified. (**H,I**) p27-mCherry cells were treated as in (F), but subsequently analyzed by time-lapse video microscopy for 24 h for their GFP (G) and mCherry (H) content in response to the transfected siRNA. (**J**) p27-mCherry cells were treated with different doses of Palbociclib to reveal and analyzed by time-lapse video microscopy.

Figure S2. Control full gel immunoblots of antibodies used in this study.
Lysates of hTERT-RPE1 cells were separated by gel eletrophoresis, immunoblotted with indicated primary antibodies (see Materials and Methods) and followed by treatment with secondary antibodies conjugated to HRP, allowed to develop and imaged on a Odyssy imaging system for the indicated amount of time (sec).

Figure S3. Localization of the p27-infliction point.
(**A**) Graphic illustrating the experimental set up. (**B**) Cells treated as in (A) are displayed based on their normalized p27-profiles. (**C**) Cells from A are continuously counted to follow population growth. (**D**) Cells from A are analyzed based on their nuclear H2B-eGFP signal to determine nuclear size over time. (**E**) Graphic illustrating the experimental set up. (**F**) Cells treated as in E are displayed based on their normalized p27-profiles. (**G**) Cells from E are continuously counted to follow population growth. (**H**) Cells from E are analyzed based on their nuclear H2B-eGFP signal to determine nuclear size over time.

Figure S4. Motif analysis of canonical and non-canonical Cdk substrate peptides identified by mass spectrometry.
(**A**) Motif analysis of all identified non-canonical Cdk phosphor-peptides. (**B**) Motif analysis of significant non-canonical Cdk phosphor-peptides. (**C**) Motif analysis of all identified canonical Cdk phosphor-peptides. (**D**) Motif analysis of significant canonical Cdk phosphor-peptides.

Supplemental Video 1. Video microscopy of p27-mCherry/H2B-GFP sensor cells transfected with non-targeting control (siCTRL) siRNA.
p27-mCherry/H2B-GFP sensor cells were transfected with non-targeting control (siCTRL) siRNA followed by time-lapse video microscopy for 48 h. mCherry and GFP signals were overlaid.

Supplemental Video 2. Video microscopy of p27-mCherry/H2B-GFP sensor cells transfected with siCdk2 siRNA for 48 h.
p27-mCherry/H2B-GFP sensor cells were transfected with siCdk2 siRNA followed by time-lapse video microscopy for 48 h. mCherry and GFP signals were overlaid.

Supplemental Video 3. Video microscopy of p27-mCherry/H2B-GFP sensor cells transfected with siRb siRNA for 48 h.
p27-mCherry/H2B-GFP sensor cells were transfected with siRb siRNA followed by time-lapse video microscopy for 48 h. mCherry and GFP signals were overlaid.

Supplemental Video 4. Video microscopy of p27-mCherry/H2B-GFP sensor cells arrested and released from Cdk4i (Palbociclib).
p27-mCherry/H2B-GFP sensor cells were treated with Cdk4i (Palbociclib) for 48 h, then released by washing and analyzed by time-lapse video microscopy over the next 22 h.

Supplemental Figure S1

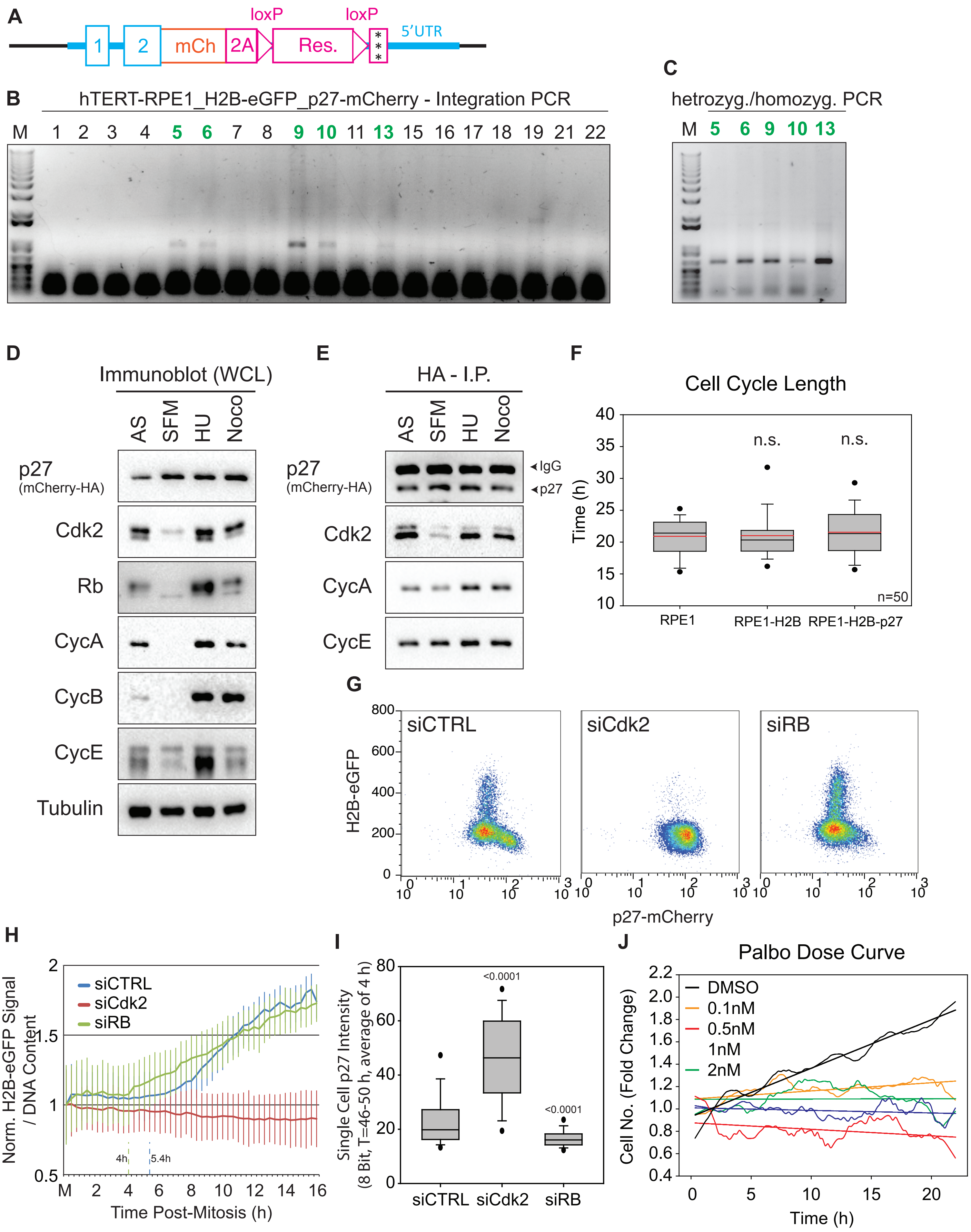

Supplemental Figure S2

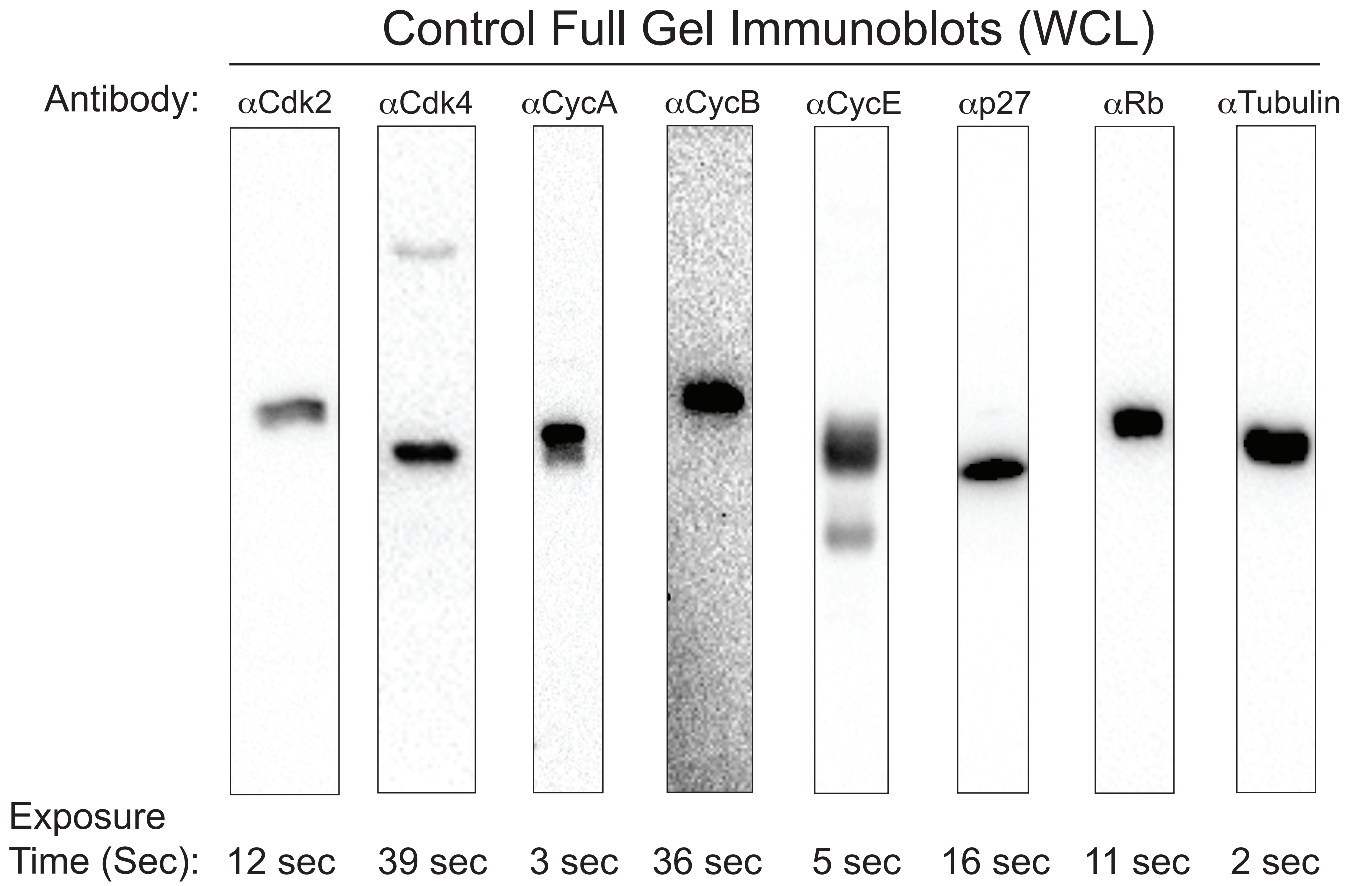

Supplemental Figure 3

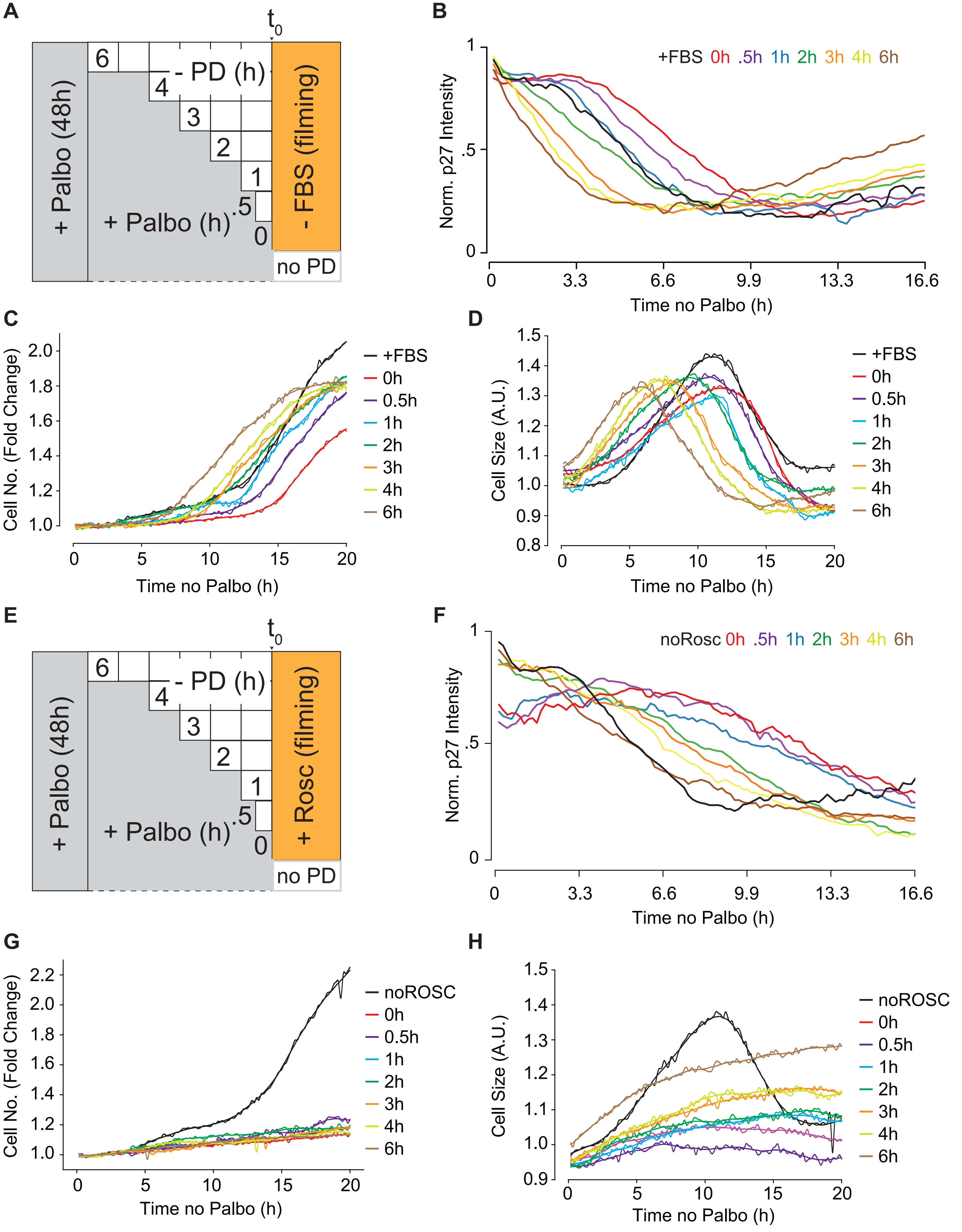

Supplemental Figure S4

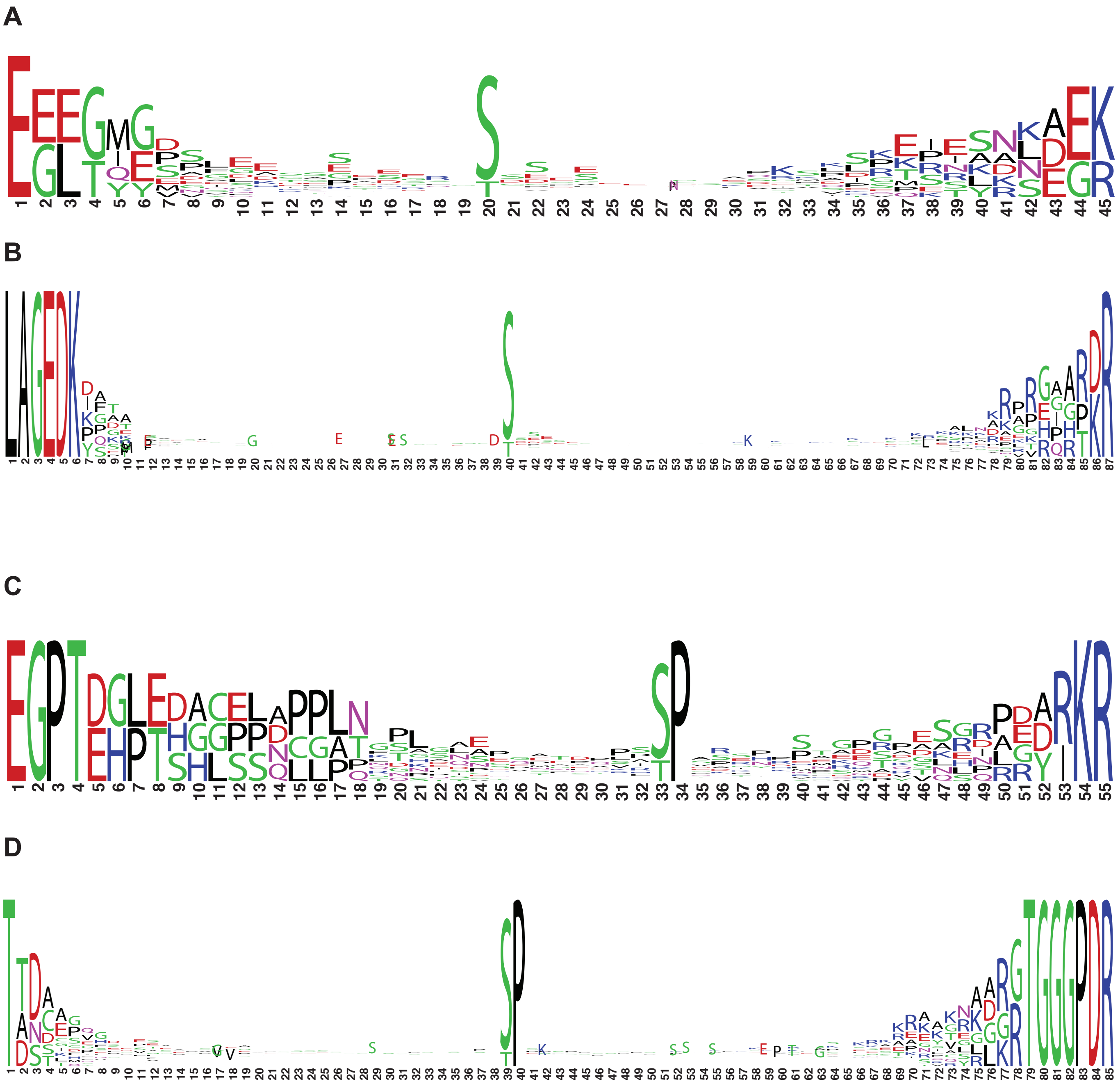

Supplement: Supplementary file 1 — Supplementary Information 1. [file 41598_2021_94200_MOESM1_ESM.pdf]
